# The genome sequence of great wood-rush, *Luzula sylvatica *(Huds) Gaudin

**DOI:** 10.12688/wellcomeopenres.20997.1

**Published:** 2024-03-01

**Authors:** Zoë A. Goodwin, David Bell, Michelle L. Hart, Peter M. Hollingsworth, Royal Botanic Garden Edinburgh Genome Acquisition Lab, Plant Genome Sizing collective, Darwin Tree of Life Barcoding collective, Wellcome Sanger Institute Tree of Life Management, Samples and Laboratory team, Wellcome Sanger Institute Scientific Operations: Sequencing Operations, Wellcome Sanger Institute Tree of Life Core Informatics team, Tree of Life Core Informatics collective, Darwin Tree of Life Consortium

**Affiliations:** 1 Royal Botanic Garden Edinburgh, Edinburgh, Scotland, UK

**Keywords:** Luzula sylvatica, great wood-rush, genome sequence, chromosomal, Poales

## Abstract

We present a genome assembly from an individual specimen of *Luzula sylvatica* (great wood-rush; Tracheophyta; Magnoliopsida; Poales; Juncaceae). The genome sequence is 444.5 megabases in span. Most of the assembly is scaffolded into 6 chromosomal pseudomolecules. The mitochondrial and plastid genome assemblies have lengths of 633.36 kilobases and 201.32 kilobases in length, respectively.

## Species taxonomy

Eukaryota; Viridiplantae; Streptophyta; Streptophytina; Embryophyta; Tracheophyta; Euphyllophyta; Spermatophyta; Magnoliopsida; Mesangiospermae; Liliopsida; Petrosaviidae; commelinids; Poales; Juncaceae; *Luzula; Luzula sylvatica* (Huds.) Gaudin (NCBI:txid59018).

## Background

The genome of great wood-rush, *Luzula sylvatica* (Huds.) Gaudin, was sequenced as part of the Darwin Tree of Life Project, a collaborative effort to sequence all named eukaryotic species in the Atlantic Archipelago of Britain and Ireland.

*Luzula sylvatica* is a densely tufted herbaceous perennial ( Stace *et al.*, 2019). It is a European temperate species and is widespread throughout Britain and Ireland, although less common in central and eastern England and central Ireland ( Stroh *et al.*, 2023). Its range in Britain and Ireland is stable, although it has declined in central and eastern England since the 1960s ( Stroh *et al.*, 2023).

It is shade tolerant and typically found in woodlands, moorlands, stream sides, and on montane ledges. It has a broad altitudinal range, from sea-level to 1040 m ( Stroh *et al.*, 2023). The flowers are hermaphrodite and observations on other *Luzula* species show that although the predominant mode of pollination is wind pollination, insect pollination may also occur ( Huang *et al.*, 2013). No hybrids of *Luzula* are recorded from Britain and Ireland ( Stace *et al.*, 2015). 

Within Britain and Ireland the species is diploid (2n = 12), with chromosome counts made on native material from three different populations in England, Ireland and Scotland ( Dempsey *et al.*, 1994; Gornall & Bailey, 1993). In this paper we present a high-quality reference genome as a foundation resource for future studies. 

## Genome sequence report

**Figure 1.**
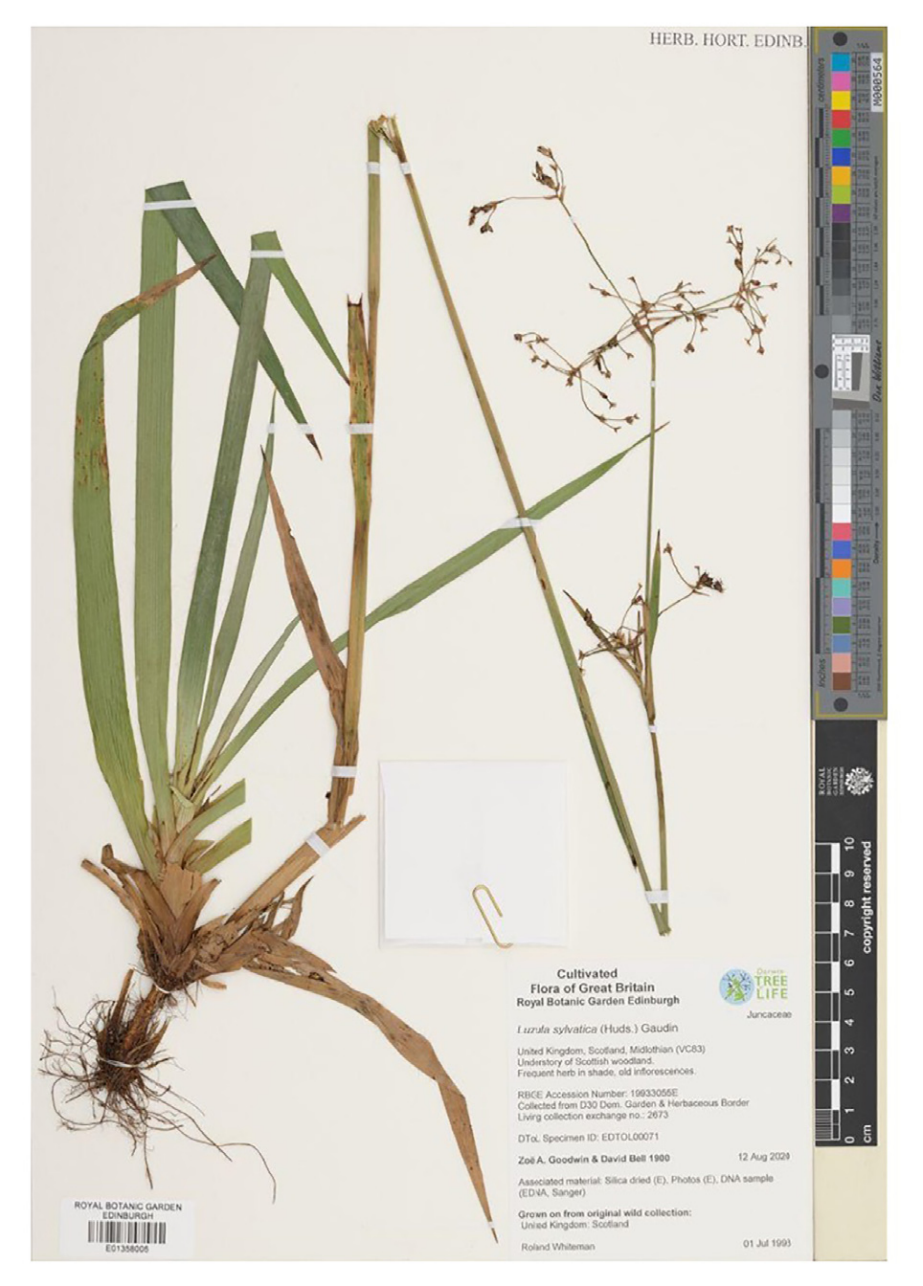
Photograph of the herbarium voucher of the *Luzula sylvatica* (lpLuzSylv1) specimen used for genome sequencing.

The genome was sequenced from *Luzula sylvatica* ( Figure 1) collected from Royal Botanic Garden Edinburgh (Inverleith), Scotland, UK (55.97, –3.21). Using flow cytometry, the genome size (1C-value) was estimated to be 0.58 pg, equivalent to 580 Mb. A total of 33-fold coverage in Pacific Biosciences single-molecule HiFi long reads was generated. Primary assembly contigs were scaffolded with chromosome conformation Hi-C data. Manual assembly curation corrected 93 missing joins or mis-joins and removed 7 haplotypic duplications, reducing the assembly length by 1.13% and the scaffold number by 50.65%, and increasing the scaffold N50 by 1.12%.

**Figure 2.**
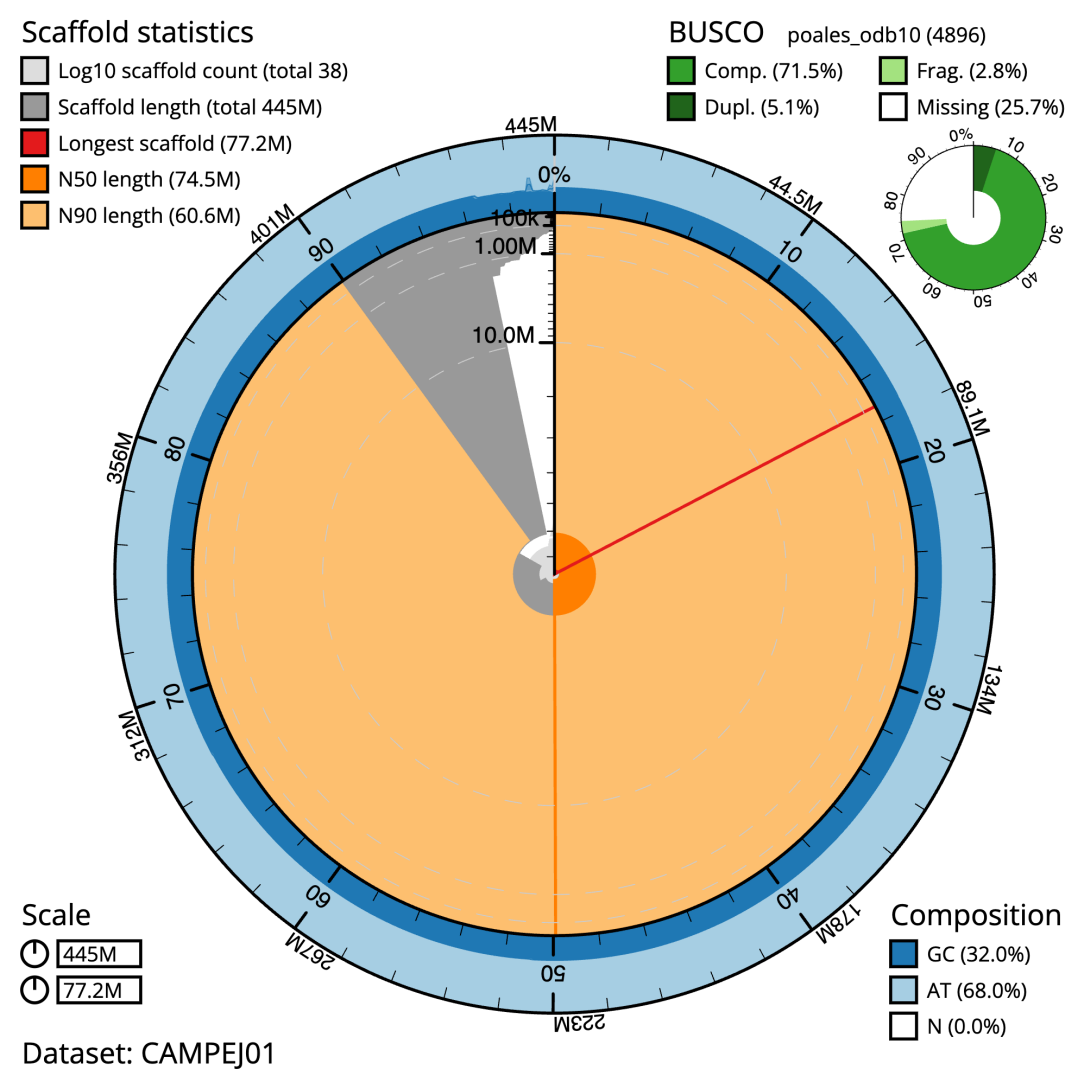
Genome assembly of *Luzula sylvatica*, lpLuzSylv1.1: metrics. The BlobToolKit Snailplot shows N50 metrics and BUSCO gene completeness. The main plot is divided into 1,000 size-ordered bins around the circumference with each bin representing 0.1% of the 445,370,405 bp assembly. The distribution of scaffold lengths is shown in dark grey with the plot radius scaled to the longest scaffold present in the assembly (77,244,432 bp, shown in red). Orange and pale-orange arcs show the N50 and N90 scaffold lengths (74,526,441 and 60,551,003 bp), respectively. The pale grey spiral shows the cumulative scaffold count on a log scale with white scale lines showing successive orders of magnitude. The blue and pale-blue area around the outside of the plot shows the distribution of GC, AT and N percentages in the same bins as the inner plot. A summary of complete, fragmented, duplicated and missing BUSCO genes in the poales_odb10 set is shown in the top right. An interactive version of this figure is available at https://blobtoolkit.genomehubs.org/view/CAMPEJ01/dataset/CAMPEJ01/snail.

**Figure 3.**
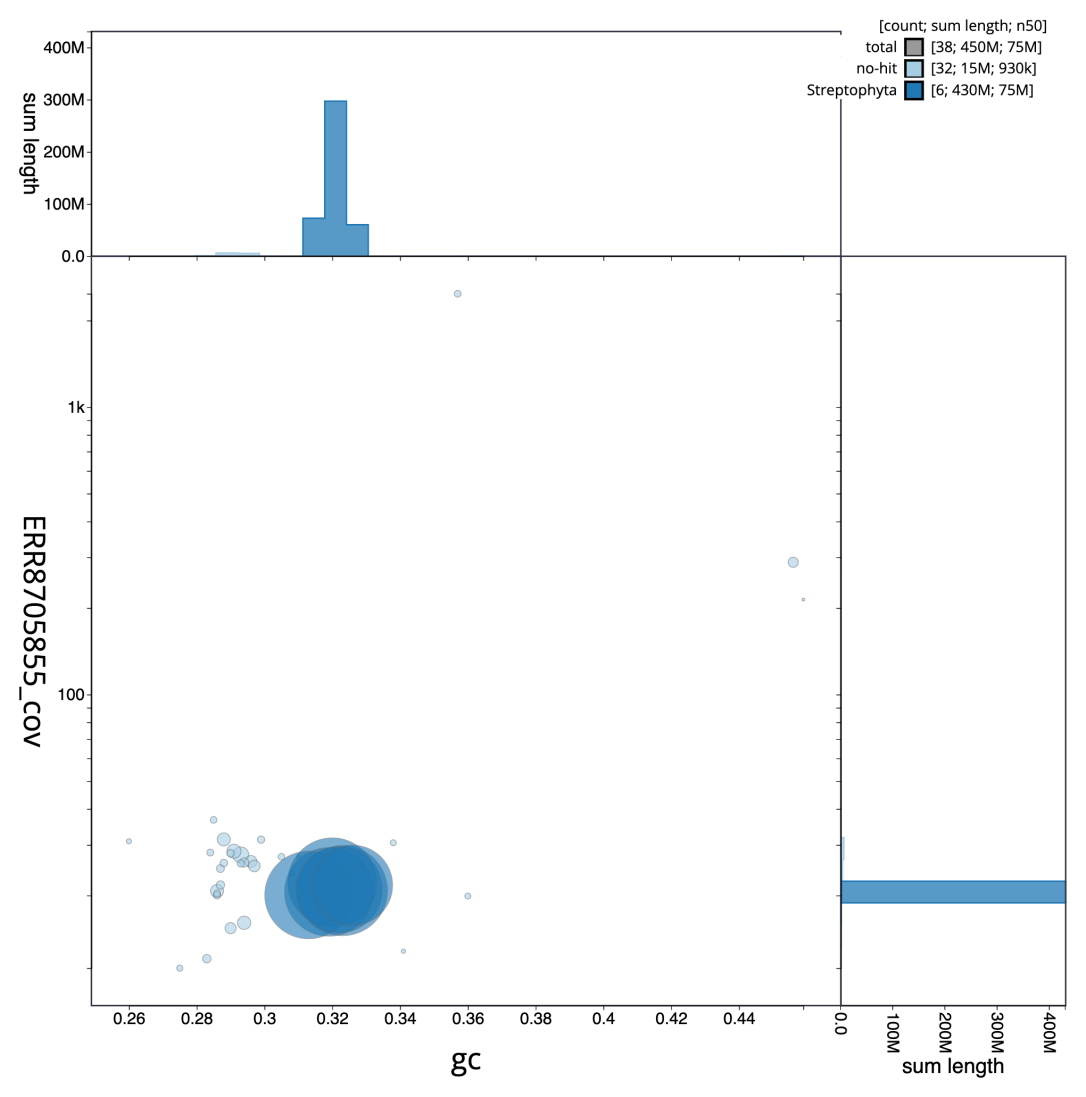
Genome assembly of *Luzula sylvatica*, lpLuzSylv1.1: BlobToolKit GC-coverage plot. Scaffolds are coloured by phylum. Circles are sized in proportion to scaffold length. Histograms show the distribution of scaffold length sum along each axis. An interactive version of this figure is available at https://blobtoolkit.genomehubs.org/view/CAMPEJ01/dataset/CAMPEJ01/blob.

**Figure 4.**
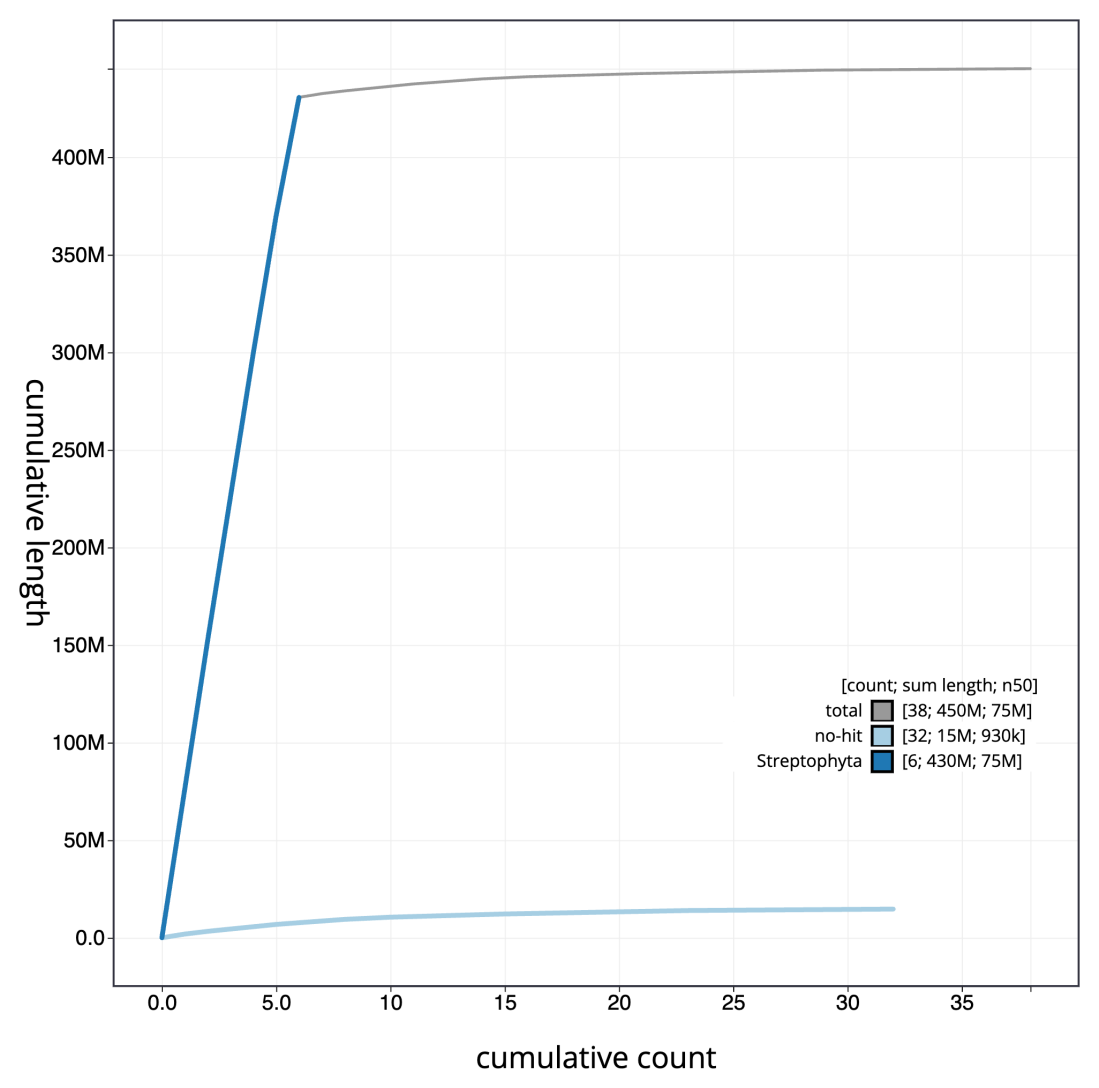
Genome assembly of *Luzula sylvatica*, lpLuzSylv1.1: BlobToolKit cumulative sequence plot. The grey line shows cumulative length for all scaffolds. Coloured lines show cumulative lengths of scaffolds assigned to each phylum using the buscogenes taxrule. An interactive version of this figure is available at https://blobtoolkit.genomehubs.org/view/CAMPEJ01/dataset/CAMPEJ01/cumulative.

**Figure 5.**
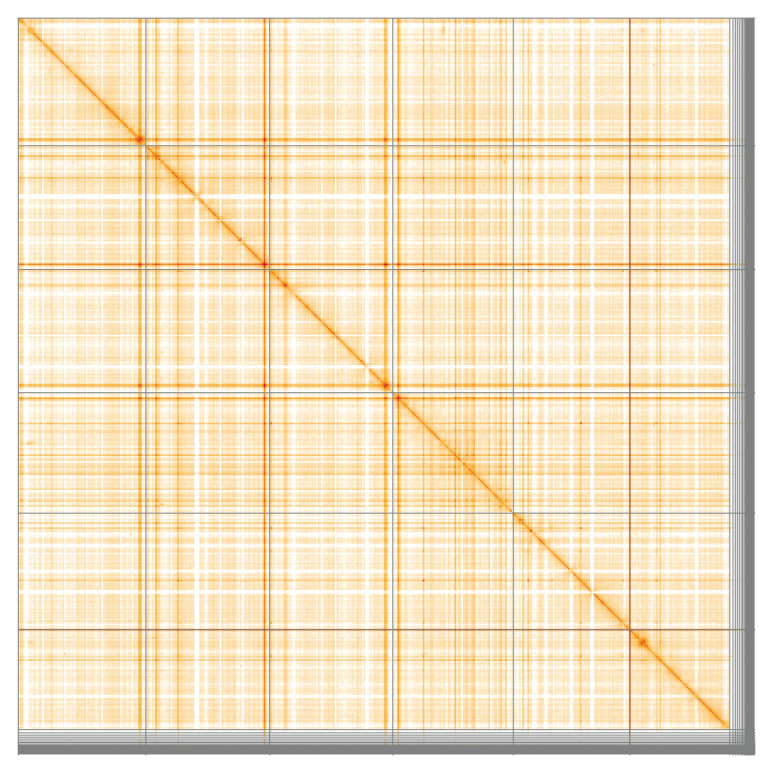
Genome assembly of *Luzula sylvatica*, lpLuzSylv1.1: Hi-C contact map of the lpLuzSylv1.1 assembly, visualised using HiGlass. Chromosomes are shown in order of size from left to right and top to bottom. An interactive version of this figure may be viewed at https://genome-note-higlass.tol.sanger.ac.uk/l/?d=OgFWtEaJQnGD2mhY8BD_Cw.

The final assembly has a total length of 444.5 Mb in 36 sequence scaffolds with a scaffold N50 of 74.5 Mb ( Table 1). The snailplot in Figure 2 provides a summary of the assembly statistics, while the distribution of assembly scaffolds on GC proportion and coverage is shown in Figure 3. The cumulative assembly plot in Figure 4 shows curves for subsets of scaffolds assigned to different phyla. Most (96.69%) of the assembly sequence was assigned to 6 chromosomal-level scaffolds. Chromosome-scale scaffolds confirmed by the Hi-C data are named in order of size ( Figure 5; Table 2). Heterozygous inversions were observed on chromosome 5, in the region of 7.3 Mb to 22.9 Mb. While not fully phased, the assembly deposited is of one haplotype. Contigs corresponding to the second haplotype have also been deposited. The mitochondrial and plastid genomes were also assembled and can be found as contigs within the multifasta file of the genome submission.

**Table 1.** Genome data for *Luzula sylvatica*, lpLuzSylv1.1.

Project accession data		
Assembly identifier	lpLuzSylv1.1	
Species	*Luzula sylvatica*	
Specimen	lpLuzSylv1	
NCBI taxonomy ID	59018	
BioProject	PRJEB50874	
BioSample ID	SAMEA7535982	
Isolate information	lpLuzSylv1: leaf tissue (DNA and Hi-C sequencing)	
Assembly metrics *		*Benchmark*
Consensus quality (QV)	59.9	*≥ 50*
*k*-mer completeness	100.0%	*≥ 95%*
BUSCO **	C:71.5%[S:66.4%,D:5.1%], F:2.8%,M:25.7%,n:4,896	*C ≥ 95%*
Percentage of assembly mapped to chromosomes	96.69%	*≥ 95%*
Sex chromosomes	-	*localised homologous pairs*
Organelles	Mitochondrial genome: 633.36 kb Plastid genome: 201.32 kb	*complete single alleles*
Raw data accessions		
PacificBiosciences SEQUEL II	ERR8705854, ERR8705855	
Hi-C Illumina	ERR8702782	
Genome assembly		
Assembly accession	GCA_946800325.1	
*Accession of * *alternate haplotype*	GCA_946800335.1	
Span (Mb)	444.5	
Number of contigs	186	
Contig N50 length (Mb)	5.3	
Number of scaffolds	36	
Scaffold N50 length (Mb)	74.5	
Longest scaffold (Mb)	77.24	

* Assembly metric benchmarks are adapted from column VGP-2020 of “Table 1: Proposed standards and metrics for defining genome assembly quality” from ( Rhie *et al.*, 2021). ** BUSCO scores based on the poales_odb10 BUSCO set using v5.3.2. C = complete [S = single copy, D = duplicated], F = fragmented, M = missing, n = number of orthologues in comparison. A full set of BUSCO scores is available at https://blobtoolkit.genomehubs.org/view/CAMPEJ01/dataset/CAMPEJ01/busco.

**Table 2.** Chromosomal pseudomolecules in the genome assembly of *Luzula sylvatica*, lpLuzSylv1.

INSDC accession	Chromosome	Length (Mb)	GC%
OX326956.1	1	77.24	32.5
OX326957.1	2	74.93	32.0
OX326958.1	3	74.53	32.0
OX326959.1	4	72.9	31.5
OX326960.1	5	70.51	32.0
OX326961.1	6	60.55	32.5
OX326962.1	MT	0.63	45.5
OX326963.1	Pltd	0.2	35.5

The estimated Quality Value (QV) of the final assembly is 59.9 with *k*-mer completeness of 100.0%, and the assembly has a BUSCO v5.3.2 completeness of 71.5% (single = 66.4%, duplicated = 5.1%), using the poales_odb10 reference set ( *n* = 4,896).

Metadata for specimens, barcode results, spectra estimates, sequencing runs, contaminants and pre-curation assembly statistics are given at https://links.tol.sanger.ac.uk/species/59018.

## Methods

### Sample acquisition, genome size estimation and nucleic acid extraction

*Luzula sylvatica* (specimen ID EDTOL00071, ToLID lpLuzSylv1) was collected from Royal Botanic Garden Edinburgh (Inverleith), Scotland, UK (latitude 55.97, longitude –3.21) on 2020-08-12. The plant was originally collected in 1993 from the Scottish highlands (Glenmore, Coire na Ciste), at an altitude of 600 m. The specimen was collected and formally identified by Zoe Goodwin and David Bell (Royal Botanic Garden Edinburgh). Leaves were cut into segments using scissors and snap-frozen in liquid nitrogen. The herbarium specimen of the sequenced plant is kept at the Royal Botanic Garden Edinburgh (E) https://data.rbge.org.uk/herb/E01358005.

The genome size was estimated by flow cytometry using the fluorochrome propidium iodide and following the ‘one-step’ method as outlined in Pellicer *et al.* (2021). For this species, the General Purpose Buffer (GPB) supplemented with 3% PVP and 0.08% (v/v) beta-mercaptoethanol was used for isolation of nuclei ( Loureiro *et al.*, 2007), and the internal calibration standard was *Solanum lycopersicum* ‘Stupiké polní rané’ with an assumed 1C-value of 968 Mb ( Doležel *et al.*, 2007).

Protocols developed by the Wellcome Sanger Institute (WSI) Tree of Life core laboratory have been deposited on protocols.io ( Denton *et al.*, 2023). The workflow for high molecular weight (HMW) DNA extraction at the WSI includes a sequence of core procedures: sample preparation; sample homogenisation, DNA extraction, fragmentation, and clean-up. In sample preparation, the lpLuzSylv1 sample was weighed and dissected on dry ice ( Jay *et al.*, 2023). HMW DNA was extracted using the Automated Plant MagAttract v2 protocol ( Todorovic *et al.*, 2023a). HMW DNA was sheared into an average fragment size of 12–20 kb in a Megaruptor 3 system with speed setting 30 ( Todorovic *et al.*, 2023b). Sheared DNA was purified by solid-phase reversible immobilisation ( Strickland *et al.*, 2023): in brief, the method employs a 1.8X ratio of AMPure PB beads to sample to eliminate shorter fragments and concentrate the DNA. The concentration of the sheared and purified DNA was assessed using a Nanodrop spectrophotometer and Qubit Fluorometer and Qubit dsDNA High Sensitivity Assay kit. Fragment size distribution was evaluated by running the sample on the FemtoPulse system.

### Sequencing

Pacific Biosciences HiFi circular consensus DNA sequencing libraries were constructed according to the manufacturers’ instructions. DNA sequencing was performed by the Scientific Operations core at the WSI on a Pacific Biosciences SEQUEL II instrument. Hi-C data were also generated from leaf tissue of lpLuzSylv1 using the Arima2 kit and sequenced on the Illumina NovaSeq 6000 instrument.

### Genome assembly, curation and evaluation

Assembly was carried out with Hifiasm ( Cheng *et al.*, 2021) and haplotypic duplication was identified and removed with purge_dups ( Guan *et al.*, 2020). The assembly was then scaffolded with Hi-C data ( Rao *et al.*, 2014) using YaHS ( Zhou *et al.*, 2023). The assembly was checked for contamination and corrected using the gEVAL system ( Chow *et al.*, 2016) as described previously ( Howe *et al.*, 2021). Manual curation was performed using gEVAL, HiGlass ( Kerpedjiev *et al.*, 2018) and Pretext ( Harry, 2022). The organelle genomes were assembled using MBG ( Rautiainen & Marschall, 2021).

A Hi-C map for the final assembly was produced using bwa-mem2 ( Vasimuddin *et al.*, 2019) in the Cooler file format ( Abdennur & Mirny, 2020). To assess the assembly metrics, the *k*-mer completeness and QV consensus quality values were calculated in Merqury ( Rhie *et al.*, 2020). This work was done using Nextflow ( Di Tommaso *et al.*, 2017) DSL2 pipelines “sanger-tol/readmapping” ( Surana *et al.*, 2023a) and “sanger-tol/genomenote” ( Surana *et al.*, 2023b). The genome was analysed within the BlobToolKit environment ( Challis *et al.*, 2020) and BUSCO scores ( Manni *et al.*, 2021; Simão *et al.*, 2015) were calculated.

Table 3 contains a list of relevant software tool versions and sources.

**Table 3.** Software tools: versions and sources.

Software tool	Version	Source
BlobToolKit	4.0.7	https://github.com/blobtoolkit/blobtoolkit
BUSCO	5.3.2	https://gitlab.com/ezlab/busco
gEVAL	N/A	https://geval.org.uk/
Hifiasm	0.16.1-r375	https://github.com/chhylp123/hifiasm
HiGlass	1.11.6	https://github.com/higlass/higlass
MBG	-	https://github.com/maickrau/MBG
Merqury	MerquryFK	https://github.com/thegenemyers/MERQURY.FK
MitoHiFi	2	https://github.com/marcelauliano/MitoHiFi
PretextView	0.2	https://github.com/wtsi-hpag/PretextView
purge_dups	1.2.3	https://github.com/dfguan/purge_dups
sanger-tol/genomenote	v1.0	https://github.com/sanger-tol/genomenote
sanger-tol/readmapping	1.1.0	https://github.com/sanger-tol/readmapping/tree/1.1.0
YaHS	yahs-1.1.91eebc2	https://github.com/c-zhou/yahs

### Wellcome Sanger Institute – Legal and Governance

The materials that have contributed to this genome note have been supplied by a Darwin Tree of Life Partner. The submission of materials by a Darwin Tree of Life Partner is subject to the **‘Darwin Tree of Life Project Sampling Code of Practice’**, which can be found in full on the Darwin Tree of Life website here. By agreeing with and signing up to the Sampling Code of Practice, the Darwin Tree of Life Partner agrees they will meet the legal and ethical requirements and standards set out within this document in respect of all samples acquired for, and supplied to, the Darwin Tree of Life Project. 

Further, the Wellcome Sanger Institute employs a process whereby due diligence is carried out proportionate to the nature of the materials themselves, and the circumstances under which they have been/are to be collected and provided for use. The purpose of this is to address and mitigate any potential legal and/or ethical implications of receipt and use of the materials as part of the research project, and to ensure that in doing so we align with best practice wherever possible. The overarching areas of consideration are:

•      Ethical review of provenance and sourcing of the material

•      Legality of collection, transfer and use (national and international) 

Each transfer of samples is further undertaken according to a Research Collaboration Agreement or Material Transfer Agreement entered into by the Darwin Tree of Life Partner, Genome Research Limited (operating as the Wellcome Sanger Institute), and in some circumstances other Darwin Tree of Life collaborators.

## Data Availability

European Nucleotide Archive: *Luzula sylvatica*. Accession number PRJEB50874; https://identifiers.org/ena.embl/PRJEB50874 ( Wellcome Sanger Institute, 2022). The genome sequence is released openly for reuse. The *Luzula sylvatica* genome sequencing initiative is part of the Darwin Tree of Life (DToL) project. All raw sequence data and the assembly have been deposited in INSDC databases. The genome will be annotated using available RNA-Seq data and presented through the Ensembl pipeline at the European Bioinformatics Institute. Raw data and assembly accession identifiers are reported in Table 1.

## Author information

Members of the Royal Botanic Garden Edinburgh Genome Acquisition Lab are listed here: https://doi.org/10.5281/zenodo.4786682.

Members of the Plant Genome Sizing collective are listed here: https://doi.org/10.5281/zenodo.7994306.

Members of the Darwin Tree of Life Barcoding collective are listed here: https://doi.org/10.5281/zenodo.4893703.

Members of the Wellcome Sanger Institute Tree of Life Management, Samples and Laboratory team are listed here: https://doi.org/10.5281/zenodo.10066175.

Members of Wellcome Sanger Institute Scientific Operations: Sequencing Operations are listed here: https://doi.org/10.5281/zenodo.10043364.

Members of the Wellcome Sanger Institute Tree of Life Core Informatics team are listed here: https://doi.org/10.5281/zenodo.10066637.

Members of the Tree of Life Core Informatics collective are listed here: https://doi.org/10.5281/zenodo.5013541.

Members of the Darwin Tree of Life Consortium are listed here: https://doi.org/10.5281/zenodo.4783558.
