# Supplementary material for: The genome sequence of great wood-rush, Luzula sylvatica (Huds) Gaudin
Source: Wellcome Open Res. 2024 Mar 1;9:124. [Version 1] doi: 10.12688/wellcomeopenres.20997.1 (PMC11380069; doi:10.12688/wellcomeopenres.20997.1)
Supplement: Peer review history

# Reviewer report: Reviewer response for version 1

**DOI:** 10.21956/wellcomeopenres.23232.r95115

**Published:** 2024-09-06

**Authors:** Yanis Bouchenak-Khelladi

**Affiliations:** 1 University of Bourgogne Franche-Comté, INRAE, Dijon, France, Dijon, France

This paper is well written, sufficiently detailed and the genome produced is of very good quality. All datasets are easily accessible and in useable format.

 I found one typo: "...sample preparation;..." use colon  instead of semicolon in the Methods_Sample acquisition section.

 Nothing else to report, it is a nice data paper.

Are sufficient details of methods and materials provided to allow replication by others?

Yes

Is the rationale for creating the dataset(s) clearly described?

Yes

Are the datasets clearly presented in a useable and accessible format?

Yes

Are the protocols appropriate and is the work technically sound?

Yes

Reviewer Expertise:

Phylogenetics, evolution, comparative analyses, eco-evolutionary dynamics

I confirm that I have read this submission and believe that I have an appropriate level of expertise to confirm that it is of an acceptable scientific standard.

# Reviewer report: Reviewer response for version 1

**DOI:** 10.21956/wellcomeopenres.23232.r89823

**Published:** 2024-08-09

**Authors:** Alemayehu Teressa Negawo

**Affiliations:** 1 Feed and Forage Development Program, International Livestock Research Institute Ethiopia, Addis Ababa, Addis Ababa, Ethiopia

The paper reads well, and present a good contribution to reference genome development for the grass spp. 

 Having said this, what is the main use of this grass? Please add a sentence or two on the importance of the crop in the introduction section. 

 In the result section, the genome assembly was presented. No genome annotation and gene family analyses were done. If possible, for better completeness of the information, please add annotation analysis.

Are sufficient details of methods and materials provided to allow replication by others?

Yes

Is the rationale for creating the dataset(s) clearly described?

Yes

Are the datasets clearly presented in a useable and accessible format?

Yes

Are the protocols appropriate and is the work technically sound?

Yes

Reviewer Expertise:

genomic/genetic analysis, plant biotechnology, data analysis, genebank, quantitative genetics, field evaluation, phenotyping

I confirm that I have read this submission and believe that I have an appropriate level of expertise to confirm that it is of an acceptable scientific standard.
